# The current state of endoscopic submucosal dissection in the UK: a nationwide cross-sectional survey

**DOI:** 10.1007/s00464-026-12596-w

**Published:** 2026-04-13

**Authors:** Said Alyacoubi, Sung Pil Hong, Nisha Patel, Amyn Haji, Mark Runciman, Ara Darzi, George Mylonas, Christopher J. Peters

**Affiliations:** 1 Department of Surgery & Cancer, Imperial College London, London, UK; 2 Imperial College Healthcare NHS Trust, London, UK; 3 King’s College Hospital NHS Foundation Trust, London, UK

**Keywords:** Endoscopic submucosal resection, Endoscopy, United Kingdom, Surveys and questionnaires

## Abstract

**Background:**

Endoscopic submucosal dissection (ESD) enables *en bloc* resection of early gastrointestinal (GI) neoplasia, but its adoption in Western countries has been limited by training and service constraints. This study aimed to describe the current landscape of ESD in the UK.

**Methods:**

A nationwide, cross-sectional, anonymised online survey was conducted among UK endoscopists independently performing either upper and/or lower GI ESD.

**Results:**

Twenty-eight responses were analysed. Most respondents were gastroenterologists (79%) and male (82%). Responses were received from across the UK, with the largest proportions from Greater London (39%) and the South East (18%). Overall, 68% had completed an advanced fellowship (commonly in the UK or Japan), and 82% had attended accredited ESD courses, often on multiple occasions. Across all ESD sites, only a minority perform more than 20 procedures annually, with high-volume practice largely confined to rectal ESD. Narrow Band Imaging (93%) and white light endoscopy (86%) were the most commonly used delineation methods; colloid-based solutions (75%), epinephrine (82%), and blue dye (100%) were widely used for submucosal injection. Most respondents routinely used tunnelling (93%) and traction-assisted techniques (82%). General anaesthesia was preferred for upper GI ESD (82%), and conscious sedation for lower GI ESD (46%). Reported barriers to training included heavy workload (37%), low caseload (26%), and limited institutional support (19%). Nearly all respondents (93%) believed robotics has a future role in ESD.

**Conclusions:**

Independent ESD practice in the UK is delivered by a small workforce, with heterogeneous training backgrounds and procedural volumes often below recommended thresholds. National strategies for structured training, centralisation, and coordinated service planning are needed to support safe and sustainable expansion of ESD.

**Graphical abstract:**

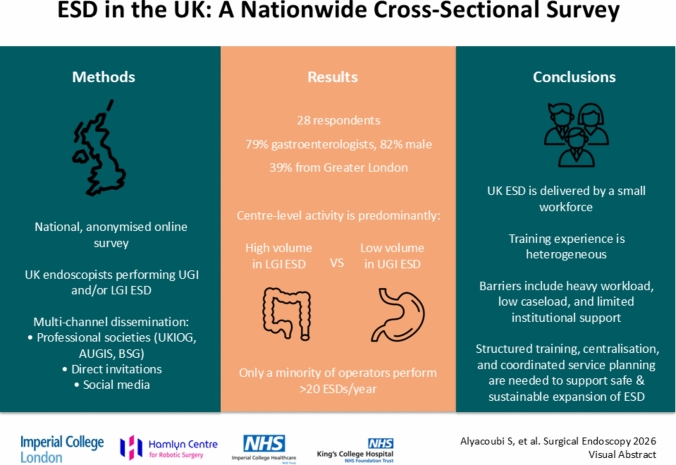

**Supplementary Information:**

The online version contains supplementary material available at 10.1007/s00464-026-12596-w.

Endoscopic submucosal dissection (ESD), developed in Japan in the 1990s, is an advanced endoscopic resection technique that enables controlled submucosal dissection for *en bloc* removal of early gastrointestinal (GI) neoplasia [1–3]. Several meta-analyses have demonstrated that, compared with endoscopic mucosal resection (EMR), ESD yields higher *en bloc* and curative resection rates with lower recurrence, irrespective of lesion size or location [4–8].

Despite accumulating evidence supporting ESD, its global implementation has varied considerably. Adoption spread rapidly across Asia, but uptake in the West has been slower, largely due to the absence of structured training pathways, limited case availability, and institutional barriers [9]. These challenges are reflected in higher *en bloc* and R0 resection rates, as well as lower complication rates, reported in Eastern centres compared with their Western counterparts [10]. Nevertheless, as expertise develops and availability improves, ESD is anticipated to gain increasing relevance in Western practice, broadening the role of endoscopic resection as a minimally invasive oncological approach [11].

Early data from single-centre studies and multicentre registry analyses support the feasibility of both upper gastrointestinal (UGI) and lower gastrointestinal (LGI) ESD in the UK [12–17]. However, beyond procedural outcomes, no nationwide, practitioner-level assessment of independent ESD practice has been performed. We therefore aimed to characterise the current landscape of independent ESD practice in the UK, including workforce characteristics, training pathways, procedural volumes, service provision, technical approaches, perceived barriers to training and service delivery, and endoscopists’ views on the potential role of robotics in ESD.

## Materials and methods

### Study design

This was a nationwide, cross-sectional, anonymised online survey conducted between 1 May and 30 June 2025. The study was designed and reported in accordance with the STROBE (Strengthening the Reporting of Observational Studies in Epidemiology) guidelines, and the completed checklist is provided as Supplementary File 1. Eligible participants were UK endoscopists independently performing either UGI or LGI ESD for precancerous or early-stage cancerous GI lesions. The survey was developed through a review of the existing literature and adaptation of relevant items from previously published surveys of ESD practice in other countries [18–21]. It was piloted with two expert ESD endoscopists to ensure clarity, relevance, and usability. The survey contained 43 items organised into five sections (Supplementary File 2): (1) demographics and professional background, (2) training and procedural experience, (3) current practice, (4) techniques and methods, and (5) logistics, training provision, and barriers. This study did not collect procedural or clinical outcome measures and was not designed to assess efficacy or safety.

### Recruitment

The survey was hosted on Qualtrics (Provo, UT, USA) and disseminated through key professional societies via email — the UK and Ireland Oesophago-Gastric (UKIOG) Cancer Group, the British Society of Gastroenterology (BSG), and the Association of Upper Gastrointestinal Surgeons of Great Britain and Ireland (AUGIS). A database of 41 independent UK ESD endoscopists, compiled by the study team with input from expert collaborators, was also used to send direct invitations and reminders. To extend its reach, the survey was promoted on X and LinkedIn by the study team and via the Institute of Global Health Innovation (IGHI) and Hamlyn Centre accounts. National accreditation datasets and professional society audits were considered; however, these sources do not provide a reliable mechanism to identify clinicians independently performing ESD or to capture practitioner-level variables required for this study.

### Ethical approval

Ethical approval was obtained from the Research Governance and Integrity Team (RGIT) at Imperial College London and the UK Health Research Authority (HRA). The study was registered with the UK Integrated Research Application System (IRAS; Project ID 354724). The study complied with the ethical standards of the Declaration of Helsinki and UK GDPR. Participation was voluntary, with electronic consent obtained before survey entry.

### Data analysis

Survey responses were analysed descriptively using the built-in Qualtrics analysis tools and then exported for further cleaning in Excel and analysis in SPSS version 30 (IBM Corp., Armonk, NY, USA). Results were reported as frequencies and percentages for categorical variables, with continuous data summarised using mean, standard deviation (SD), median, and interquartile range (IQR). Where appropriate, categorical responses were collapsed into broader groups to aid interpretation. Given the exploratory nature of the study and the small national pool of independent ESD practitioners, no formal a priori sample size or power calculation was performed. As no lesion- or patient-level outcomes were collected, inferential analyses and hypothesis testing were not undertaken to avoid overinterpretation of a small, self-reported dataset not designed for causal inference.

## Results

### Response rate

A total of 28 eligible responses were received: 27 were fully completed, and one was 74% complete. All 28 responses were included in the analysis.

### Demographics and professional background

Of the 28 respondents, the majority were aged 40–59 years (24; 85.7%) and were male (23; 82.1%). Responses were received from across the UK, with Greater London accounting for the largest proportion (11; 39.3%). Most respondents were gastroenterologists (22; 78.6%), while the remainder were UGI or LGI surgeons (3; 10.7% each). Most respondents had substantial consultant experience, with 18 (64.3%) reporting more than 10 years in practice. Table 1 summarises respondent demographics and professional characteristics.

**Table 1:** Summary of respondent characteristics (*N* = 28)

Variable	Category	*n* (%)
Age	40–49 years	15 (53.6)
	50–59 years	9 (32.1)
	60 + years	4 (14.3)
Gender	Male	23 (82.1)
	Female	5 (17.9)
Region	Greater London	11 (39.3)
	South East (not including London)	5 (17.9)
	Wales	3 (10.7)
	North East	2 (7.1)
	East of England	2 (7.1)
	South West	1 (3.6)
	North West	1 (3.6)
	East Midlands	1 (3.6)
	Scotland	1 (3.6)
	Northern Ireland	1 (3.6)
	Yorkshire and the Humber	0
	West Midlands	0
Speciality	Gastroenterologist	22 (78.6)
	UGI surgeon	3 (10.7)
	LGI surgeon	3 (10.7)
Years as consultant	< 5 years	3 (10.7)
	5–10 years	7 (25)
	11–20 years	11 (39.3)
	> 20 years	7 (25)

### Training and procedural experience in ESD

Nineteen respondents (67.9%) reported having completed an advanced endoscopy fellowship, while 9 (32.1%) had not. Among those with fellowships (*n* = 19), the UK was the most common location (8; 42.1%), followed by Japan (5; 26.3%), South Korea (2; 10.5%), China (2; 10.5%), and Canada (1; 5.3%). One respondent (5.3%) reported having completed fellowships in both the UK and Japan. Fellowship durations varied: 6 participants (31.6%) trained for < 6 months, 6 (31.6%) for 6–12 months, 3 (15.8%) for 1–2 years, and 4 (21.1%) for more than 2 years. Of the 19 participants, 14 (73.7%) confirmed their fellowship included specific ESD training, while 5 (26.3%) reported it did not.

Nearly all participants (23/28; 82.1%) had received formal training in ESD via accredited courses or workshops, often attending multiple sessions: 13 (56.5%) attended > 5 courses, 4 (17.4%) attended 4–5, 4 (17.4%) attended 2–3, and 2 (8.7%) attended 1. Training through mentorship was also common: 24 participants (85.7%) reported receiving guidance from experienced endoscopists. Among those receiving mentorship (*n* = 24), the most common formats were observation of expert procedures (21; 87.5%), case discussions and feedback (19; 79.2%), and direct hands-on supervision (17; 70.8%).

Overall procedural experience during ESD training varied across ex vivo, in vivo*,* and human cases, with full details shown in Table 2**.**

**Table 2:** Number of procedures completed during ESD training (*N* = 28)

Procedure type	0	1–5	6–10	11–20	21–30	> 30
Ex vivo ESD	2	5	4	6	5	6
In vivo (live pig) ESD	8	7	8	3	0	2
Human ESD (assistant-supervised)	5	6	5	3	4	5
Human ESD (primary operator-supervised)	5	5	8	2	2	6

### Current ESD practice

Respondents reported variable numbers of independent ESD practitioners in their centres, most commonly 1–3, accounting for 82.1% of responses. Smaller numbers reported four practitioners (4; 14.3%) or five (1; 3.6%). As these are individual reports, multiple responses may originate from the same centre and should not be interpreted as unique institutional totals**.** Duration of independent ESD practice also varied widely, ranging from 1 to 19 years (mean 9, SD 5.6; median 8, IQR 4–15). One respondent (3.6%) did not provide this information. When categorised, over two-thirds had ≥ 6 years’ experience, including 10 (35.7%) with > 10 years (highly experienced) and 9 (32.1%) with 6–10 years (intermediate).

For descriptive purposes, centre-level ESD activity was categorised as low (< 20/year), medium (20–50/year), or high (> 50/year), while individual activity was defined as low (< 10/year), medium (10–20/year), and high (> 20/year). Although no universal standard exists for Western ESD, these thresholds were chosen pragmatically to align with European data (e.g., German ESD registry: ≤ 20, 20–50, > 50 cases/year) and the European Society of Gastrointestinal Endoscopy (ESGE) guidance recommending ≥ 25 procedures annually to maintain proficiency [22, 23]. These cut-offs therefore provide clinically meaningful, context-appropriate groupings. Full details are presented in Table 3**.**

**Table 3:** ESD procedure volumes as reported by respondents (*N* = 28)^a^

A. Categorised annual ESD volumes per centre by anatomical site				
Anatomical site	Low, *n* (%)	Medium, *n* (%)	High,* n* (%)	NA^b^, *n* (%)
	< 20/year	20–50/year	> 50/year	
UGI ESD	14 (50.0%)	5 (17.9%)	5 (17.9%)	4 (14.3%)
LGI ESD	8 (28.6%)	3 (10.7%)	14 (50.0%)	3 (10.7%)

B. Categorised annual ESD volumes per respondent by anatomical site				
Anatomical site	Low, *n* (%)	Medium, *n* (%)	High, *n* (%)	NA^b^, *n* (%)
	< 10/year	10–20/year	> 20/year	
Oesophagus	8 (28.6%)	4 (14.3%)	5 (17.9%)	11 (39.3%)
Stomach	14 (50%)	5 (17.9%)	2 (7.1%)	7 (25.0%)
Duodenum	10 (35.7%)	1 (3.6%)	0 (0%)	17 (60.7%)
Colon	4 (14.3%)	6 (21.4%)	8 (28.6%)	10 (35.7%)
Rectum	3 (10.7%)	3 (10.7%)	14 (50%)	8 (28.6%)

^a^Centre identifiers were not collected; therefore, multiple respondents may have reported from the same institution, and figures reflect individual perceptions rather than definitive institutional activity

^b^*NA* not applicable, respondents did not provide this information, likely indicating they do not perform this procedure

Based on respondent reports, half (50%) indicated that their centres performed fewer than 20 UGI ESDs per year, while 18% reported more than 50 cases annually. LGI ESD activity was higher, with half of respondents (50%) indicating that their centres performed more than 50 procedures per year and 29% reporting fewer than 20 cases (Table 3A).

At the individual level, annual ESD volumes varied across anatomical sites. Rectal ESD showed the highest procedural activity, with half of respondents (50%) performing more than 20 resections per year. Colonic ESD activity was more evenly distributed, with 29% performing more than 20 procedures annually. UGI ESD volumes were lower: 18% of respondents performed more than 20 oesophageal cases per year, and 7% exceeded this threshold for gastric ESD. Most UGI practitioners performed fewer than 10 procedures annually. Duodenal ESD was rarely performed, with no respondent reporting more than 10 cases per year (Table 3B).

When examined by specialty, gastroenterologists (*n* = 22) reported the widest range of ESD activity, performing procedures across all GI sites. Most carried out gastric and rectal ESD (17 respondents each), followed by colonic (16), oesophageal (12), and duodenal (9). When categorised by anatomical focus, over half of gastroenterologists (54.5%) performed both UGI and LGI ESDs, while smaller proportions specialised exclusively in UGI or LGI procedures (22.7% each). All three UGI surgeons performed oesophageal and gastric ESD, with one also undertaking duodenal procedures. Among the three LGI surgeons, all performed rectal ESD, and the same two who performed colonic ESD also reported some UGI activity.

Among the 28 respondents, UGI ESD was most performed in the operating theatre (14; 50.0%), followed by the endoscopy unit (6; 21.4%) or both locations (4; 14.3%). For LGI ESD, most procedures were performed in the endoscopy unit (13; 46.4%), with 5 (17.9%) reporting both settings and 2 (7.1%) exclusively in the operating theatre. General anaesthesia (GA) was the predominant sedation method for UGI ESD (23; 82.1%). In contrast, LGI ESD was most often performed under conscious sedation with opioids and benzodiazepines (13; 46.4%), followed by monitored anaesthesia care (MAC) with propofol (4; 14.3%) and GA (3; 10.7%).

### ESD techniques and methods

Respondents reported using a variety of lesion delineation techniques, most commonly Narrow Band Imaging (NBI) (26; 92.9%) and White Light Endoscopy (WLE) (24; 85.7%). For submucosal injection, colloid solutions such as Hydroxyethyl Starch (HES) and Succinylated Gelatine were most frequently used (21; 75.0%). Most respondents (23; 82.1%) routinely added epinephrine, and all respondents (28; 100%) incorporated a blue dye (indigo carmine or methylene blue) into the submucosal injection solution.

Regarding the knives used, the DualKnife/J (Olympus, Tokyo, Japan) was the most frequently reported (20; 71.4%), followed by the IT Knife family (Olympus, Tokyo, Japan) (16; 57.1%) and the FlushKnife family (Fujifilm, Tokyo, Japan) (12; 42.9%). Among dissection techniques, the tunnelling method was most frequently reported (26; 92.9%), followed by traction-assisted ESD (23; 82.1%) and conventional ESD (21; 75.0%) (see Table 4 for full details on practice patterns among respondents).

**Table 4:** Summary of ESD practice patterns among respondents (*N* = 28)

Domain	Item	*n* (%)
Lesion delineation	Narrow band imaging (NBI)	26 (92.9%)
	White light endoscopy (WLE)	24 (85.7%)
	Indigo carmine chromoendoscopy	13 (46.4%)
	Lugol’s iodine chromoendoscopy	10 (35.7%)
	Acetic acid chromoendoscopy	8 (28.6%)
	Blue light imaging (BLI)	6 (21.4%)
	Linked colour imaging (LCI)	5 (17.9%)
	Endoscopic ultrasound	3 (10.7%)
	Crystal violet chromoendoscopy	1 (3.6%)
	Texture and colour enhancement imaging (TXI)	1 (3.6%)
Injection solution	Colloid solutions (HES, Gelatine)	21 (75.0%)
	Normal saline (NaCl 0.9%)	9 (32.1%)
	Eleview®	8 (28.6%)
	Dextrose water	2 (7.1%)
	Hyaluronic acid	2 (7.1%)
	EverLift®	1 (3.6%)
Adjuvants	Epinephrine	23 (82.1%)
	Blue dye	28 (100%)
ESD knives	DualKnife/J (Olympus)	20 (71.4%)
	IT knife family^a^ (Olympus)	16 (57.1%)
	Flushknife family^b^ (Fujifilm)	12 (42.9%)
	Speedboat knife (Creo Medical)	6 (21.4%)
	Hybridknife family^c^ (ERBE)	4 (14.3%)
	Splashknife (pentax Medical)	3 (10.7%)
	Clutchcutter (Fujifilm)	2 (7.1%)
	Hookknife/J (Olympus)	1 (3.6%)
	SB knife (Olympus)	1 (3.6%)
ESD techniques	Tunnelling ESD (Tu-ESD)	26 (92.9%)
	Traction-assisted ESD (T-ESD)	23 (82.1%)
	Conventional ESD (C-ESD)	21 (75.0%)
	Pocket-creation method^d^ (PCM)	17 (60.7%)
	Hybrid ESD	11 (39.3%)
	Underwater ESD	8 (28.6%)
	Endoscopic intermuscular dissection (EID)	6 (21.4%)

^a^Includes IT knife, IT knife 2, and IT knife nano variants

^b^Includes flushknife *BT* (ball-tip) and flushknife *NS* (needle-shaped) variants

^c^Includes hybridknife I-type, Hybridknife T-type, and Hybridknife flex I-type 1.5 mm variants

^d^Includes SITE and double-pocket butterfly variants

All respondents (28; 100%) reported using a distal attachment cap, and the majority (25; 89.3%) employed traction techniques. Among these, the most common was the clip-and-line method (10 of 25; 40.0%), followed by double-clip techniques (6 of 25; 24.0%), internal traction wires such as the ProdiGI Traction Wire (5 of 25; 20.0%), and clip-and-band or clip-and-snare approaches (2 of 25 each; 8.0%).

Among 28 respondents, familiarity with emerging robotic endoscopic systems to assist ESD was limited, with only 11 respondents (39.3%) reporting awareness. The only systems mentioned were the MASTER (Nanyang Technological University, Singapore) and EndoMaster EASE (EndoMaster Pte Ltd, Singapore).

Subsequent analyses reflect responses from 27 participants, as one questionnaire was incomplete. Despite this, most agreed that robotics have a role in ESD (25; 92.6%), and nearly as many expressed interest in adopting them (24; 88.9%).

When ranking desirable features of a robotic-endoscopic platform, adjustable or dynamic traction scored highest (189), followed by independent motion of the scope and traction device (177) and operative site stabilisation (149). Interchangeable instruments and endoscope compatibility were rated equally (131), while ease of insertion (75), low footprint (68), and dual UGI/LGI applicability (51) ranked lower.

### Logistics, training provision, and barriers

Of 27 respondents, 11 (40.7%) reported that securing endoscopy time was easy/very easy, 7 (25.9%) found it difficult/very difficult, and 9 (33.3%) were neutral. Equipment availability was reported as easy/very easy by 18 (66.7%), difficult by 6 (22.2%), and neutral by 3 (11.1%). Access to anaesthesia support showed greater variability, with 8 (29.6%) rating it easy, 11 (40.7%) difficult or very difficult, and 8 (29.6%) neutral.

Waiting times were generally short: 8 (29.6%) reported 2–4 weeks, and 15 (55.6%) reported 1–3 months. Longer delays were less common, with 3 (11.1%) reporting 3–6 months and 1 (3.7%) reporting 7–12 months. Prospective data collection was widespread, with 23 (85.2%) maintaining an ESD database.

Nearly two-thirds (17; 63.0%) reported that their institutions offered ESD mentorship for internal and/or external consultants. A quarter (7; 25.9%) reported no mentorship provision, while 3 (11.1%) indicated that such programmes were under consideration. Fellowship opportunities varied: 7 (25.9%) reported structured national or international programmes, 6 (22.2%) local programmes, 6 (22.2%) none, and 8 (29.6%) centres developing or exploring fellowship schemes. Hands-on training for in-house gastroenterology or surgical trainees was available in half of the centres (14; 51.9%).

The most frequently cited challenges to ESD training were heavy workload (10; 37.0%) and low ESD case volume (7; 25.9%). Other barriers included limited institutional support (5; 18.5%) and absence of a formal training programme (3; 11.1%). Only two respondents (7.4%) identified a lack of suitable cases as the primary barrier.

## Discussion

This study provides a national overview of independent ESD practice in the UK. Although no central registry exists, informal expert consultation suggests a national ESD workforce of approximately 40–45 clinicians. Our multipronged dissemination strategy was therefore designed to maximise reach within this group. Responses were geographically widespread and likely capture a substantial proportion of the active ESD workforce. The respondent number (*n* = 28) is comparable to similar national surveys from Canada and Italy (20–30 respondents), reflecting the limited scale of Western ESD practice [18–20]. In contrast, a recent Korean survey reported 68 responses from 31 hospitals, consistent with a larger workforce and wider adoption of ESD in East Asia [21].

ESD practice in the UK appears concentrated among a small group of endoscopists, who are likely working in specialist or tertiary referral centres, reflecting both limited case availability and the high level of expertise required for independent practice. The underrepresentation of women mirrors broader gender disparities in interventional endoscopy [24, 25], while the limited involvement of surgeons aligns with international trends showing that ESD remains largely gastroenterologist-led [19, 20].

Training experience was highly variable. Although most respondents had completed an advanced endoscopy fellowship, exposure to ESD within these programmes was inconsistent. Consequently, skill acquisition appears to rely heavily on supplementary mechanisms, including repeated course attendance and informal mentorship from experienced operators. These findings suggest that ESD competency in the UK is achieved through prolonged, iterative exposure rather than structured progression within a standardised national training pathway.

Reported annual ESD volumes varied. At the institutional level, most units were perceived as low volume for UGI ESD, whereas LGI activity was more commonly reported as high volume. Contextualising these findings, large-scale database studies have demonstrated an association between higher hospital volumes and fewer ESD-related complications, highlighting potential concerns around low-volume practice [26, 27]. Recent European multicentre data reinforce this relationship but also demonstrate that acceptable outcomes can be achieved in lower-volume units when supported by structured referral systems, mentorship, and careful case selection—underscoring the importance of organised service pathways alongside centralisation [22, 28].

At the individual level, rectal ESD emerged as the most established site, with most active operators reporting high activity. Colonic ESD showed more variable engagement, while oesophageal and gastric ESD were typically performed at low annual volumes; duodenal ESD was rarely undertaken. Only a minority of respondents achieved high procedural volumes (> 20 cases per year), ranging from 7% for gastric to 50% for rectal ESD, indicating that few operators meet ESGE-recommended proficiency thresholds, and that high-volume ESD activity in the UK remains largely confined to the colorectum, particularly the rectum.

The scale of ESD practice in the UK must be interpreted in the context of disease epidemiology, diagnostic pathways, and service organisation. Gastric cancer—historically the principal driver of ESD development in East Asia—has a substantially lower incidence in the UK, and the absence of population-based screening results in fewer early lesions suitable for ESD [29, 30]. Although colorectal cancer incidence in the UK is high and a national FIT-based screening programme exists, only a small proportion of detected lesions are amenable to ESD. Established modalities such as EMR and surgical resection remain embedded within national guidelines, with ESD reserved for selected cases [31–33]. Moreover, the technical complexity and prolonged learning curve of LGI ESD, together with early Western outcome data less favourable than those from Eastern centres, contributed to historical scepticism among Western endoscopists [32, 34]. These demand-side and system-level factors help explain the relatively small UK ESD workforce and lower procedural volumes.

While core practices of ESD were broadly consistent, substantial heterogeneity was observed in dissection strategies and traction techniques, reflecting the absence of consensus on optimal technical approaches. The predominance of traction-assisted dissection suggests increasing adoption of approaches that may improve safety and efficiency, while the diversity of traction methods highlights both their perceived value and lack of standardisation. This variability underscores an unmet need for tools that can provide reproducible, controllable traction, creating opportunities for innovation, including dedicated traction devices and robotic platforms [35, 36].

Awareness of robotic ESD systems among respondents was limited, reflecting their predominantly experimental or preclinical status. Nevertheless, most recognised a potential role for robotics and expressed interest in adoption if available, particularly to address challenges related to dynamic traction, independent scope–device motion and working site stabilisation. While emerging robotic platforms aim to address these limitations [37, 38], their clinical feasibility, cost–benefit balance, and integration into routine practice remain uncertain and beyond the scope of this study.

Logistical and institutional constraints remain key challenges to ESD delivery and training in the UK, including variable access to endoscopy time, equipment, and anaesthesia support. Heavy clinical workloads and limited case availability further restrict both training opportunities and routine practice. These barriers appear primarily structural rather than individual, compounding the existing technical complexity and learning-curve challenges. Failure to address these constraints risks delayed access to curative endoscopic therapy and variability in procedural safety. These findings reinforce the need for system-level strategies to secure resources, formalise training, and prioritise ESD services, as highlighted by Barbour et al. (2020) [39] and Araújo-Martins et al. (2019) [40]. Consequently, effective solutions are more likely to lie in service organisation—such as protected endoscopy time, structured referral pathways, and centralised expertise—than in individual operator factors alone.

This study has important implications for clinical practice and service delivery. High-volume centres are best positioned to deliver complex ESD and serve as hubs for workforce development through structured training, fellowships, and mentorship. Practice in lower-volume centres should be embedded within structured regional referral networks, with careful case selection and clear pathways for mentorship and escalation of difficult cases. The observed heterogeneity in training pathways highlights the need for a nationally standardised, longitudinal, competency-based approach to ESD training, with staged progression from preclinical training and observation of human cases to supervised clinical practice and independent practice once competency is demonstrated. The widespread use of prospective ESD databases reflects a strong culture of audit and outcome monitoring; expansion of systematic use of registry data could support quality assurance, benchmarking, workforce planning, and phased service expansion.

This study has several limitations. First, the number of respondents was small, and some survey items were generic, limiting detailed subgroup analysis. Second, the survey targeted independent ESD practitioners and therefore does not capture trainee perspectives. Third, all data were self-reported and thus subject to recall bias, particularly for procedural volumes and training histories. Fourth, although the demographics and geographical distribution likely reflect the current composition and centralised structure of the UK ESD workforce, it limits extrapolation beyond this cohort. Finally, anonymisation and the absence of centre identifiers precluded institutional-level analyses, meaning that findings primarily reflect individual practitioner perspectives rather than formal comparisons between centres or service models.

In summary, ESD in the UK remains in an early phase of system-level dissemination, with important implications for training, service provision, and future expansion. While further expansion of ESD services will require national coordination, pragmatic and incremental approaches—anchored in structured training, centralisation and service planning—may represent the most feasible route to safe and sustainable service development within current resource constraints.

## Supplementary Information

Below is the link to the electronic supplementary material.

Supplementary file1 (DOCX 44 KB)

Supplementary file2 (PDF 415 KB)
