# Correction: The current state of endoscopic submucosal dissection in the UK: a nationwide cross-sectional survey

**DOI:** 10.1007/s00464-026-12990-4

**Published:** 2026-06-02

**Authors:** Said Alyacoubi, Sung Pil Hong, Nisha Patel, Amyn Haji, Mark Runciman, Ara Darzi, George Mylonas, Christopher J. Peters

**Affiliations:** 1 Department of Surgery & Cancer, Imperial College London, London, UK; 2 Imperial College Healthcare NHS Trust, London, UK; 3 King’s College Hospital NHS Foundation Trust, London, UK

**Corrrection to: Surgical Endoscopy**

10.1007/s00464-026-12596-w

The original online version of this article was revised to correct a reference citation in the Discussion section. The correct citation is as follows:

In contrast, a recent Korean survey reported 68 responses from 31 hospitals, consistent with a larger workforce and wider adoption of ESD in East Asia [21].

The original article has been corrected.

