# Supplementary material for: The current state of endoscopic submucosal dissection in the UK: a nationwide cross-sectional survey
Source: Surg Endosc. 2026 Apr 13;40(7):5713–21. doi: 10.1007/s00464-026-12596-w (PMC13369237; doi:10.1007/s00464-026-12596-w)
Supplement: Supplementary file 2 — Supplementary file2 (PDF 415 KB) [file 464_2026_12596_MOESM2_ESM.pdf]

.

Endoscopic Submucosal Dissection (ESD) in the UK: A Survey of Training Pathways and Practice Trends

Rationale:

Current endoscopic techniques used to treat early-stage gastrointestinal (GI) cancer include endoscopic mucosal resection (EMR) and endoscopic submucosal dissection (ESD). ESD has been shown to offer superior outcomes in terms of en bloc resection rates, negative surgical margins, and recurrence rates. However, ESD is predominantly practised by highly skilled experts given its steep learning curve and the advanced dexterity it requires. Despite the increasing adoption of ESD in the UK, there is limited data on the training pathways and scope of ESD practice. Understanding current practices will help identify barriers, training requirements, and opportunities for standardisation of ESD practice in the UK.

Invitation:

You are invited to participate in this online survey study, which aims to assess the current landscape of ESD practice in the UK, including training, techniques, and barriers to implementation.

This survey consists of five sections:

1. Demographics and Professional Background

2. Training in ESD

3. Current ESD Practice

4. ESD Techniques and Methods

5. Barriers and Challenges

Your responses will be collected using Qualtrics, which complies with the UK General Data Protection Regulation (UK GDPR). All data will be securely stored and used exclusively for research purposes. Findings from this study may be published in academic journals or presented at conferences.

Frequently Asked Questions (FAQs):

Do I have to take part?

No, participation in this study is entirely voluntary. If you choose to participate, you can still exit the survey at any time before submission. However, as the survey is anonymous, once you have submitted your responses, it will not be possible to withdraw your data.

What will happen to me if I take part?

You will be asked to complete an anonymous online survey about your ESD training, practice, and challenges. The survey takes approximately 15–20 minutes. You can save your progress and return to complete the survey later using the same link. Your responses will be securely stored and used solely for research purposes. No further involvement is required after submission.

What do I have to do?

Simply complete the 15–20-minute online survey, answering questions about your background, ESD training, current practice, techniques, and any challenges you face.

What are the possible benefits of taking part?

- By participating, you will contribute to a better understanding of ESD training pathways and practice trends in the UK.
- The findings may help inform future training programmes, standardisation efforts, and policy development for ESD.

- The study results will be disseminated to relevant professional societies, potentially leading to enhanced training opportunities and professional development in the field.

What are the possible disadvantages of taking part?

- The survey will take approximately 15–20 minutes to complete, which may be an inconvenience.
- There are no direct personal benefits for participants, as no remuneration is provided.
- Since responses are anonymous, you will not be able to withdraw your data after submission.

What will happen to the results of the research study?

The findings will be analysed and may be published in academic journals or presented at conferences. Results will also be shared with relevant professional medical societies to help inform ESD training and practice in the UK. All data will remain anonymous, and individual participants will not be identifiable in any publications or reports.

Who is organising and funding the research?

This study is being conducted as part of a PhD research project at Imperial College London. This study requires no funding, and the researchers declare no conflicts of interest.

Who has reviewed the study?

This study has been reviewed and approved by the academic supervisors of the PhD student and the Research Governance and Integrity Team (RGIT) at Imperial College London in accordance with institutional research governance procedures. The study has obtained ethical approval from Health Research Authority (HRA). If you have any questions about the study's ethical approval, you may contact the research team or the relevant ethics office at Imperial College London.

Contact for further information:

Mr Said Alyacoubi MD MSc (Oxon) MRCS (Eng)

Clinical Research Fellow and PhD Student – Department of Surgery and Cancer, Imperial College London

Email: S.alyacoubi24@imperial.ac.uk

.

Eligibility:

To participate in this study, you must be:

- A UK-based endoscopist **AND**
- Currently perform Endoscopic Submucosal Dissection (ESD) in the UK

. **Do you meet the eligibility criteria?**

○ Yes

○ No

.

Consent Statement:

- I confirm that I have read and understood the study and participant information presented at the beginning of this survey.
- I understand that my participation is voluntary, and I am free to withdraw at any time, without giving any reason and without my legal rights being affected.
- I understand that data collected from me are a gift donated to Imperial College London and that I will not personally benefit financially if this research leads to an invention and/or the successful development of a new test, device, medication, product or service.

. **I consent to participate in this study**

☐ I consent
☐ I don't consent

Block 1

. A: Demographics and Professional Background

. What is your age range?

○ 20-29

- 30-39
- 40-49
- 50-59
- 60+

. What is your gender?

- Male
- Female
- Prefer not to say
- Other (please specify)

. In which UK region do you primarily practice?

- Wales
- Scotland
- Northern Ireland
- Greater London
- Yorkshire
- East Midlands
- West Midlands
- North East
- North West
- South East
- South West
- East of England

. What is your speciality?

- Gastroenterologist
- Upper GI Surgeon
- Lower GI Surgeon

. How many years have you been practising as a consultant?

- Less than 5 years
- 5–10 years
- 11–20 years
- More than 20 years

Block 2

. **B: Training in ESD**

. Did you complete an advanced endoscopy fellowship?

- Yes
- No

. Where did you complete your advanced endoscopy fellowship?

- UK
- Europe
- USA
- Canada
- Japan
- [ ] Other (please specify)

. How long was your advanced endoscopy fellowship?

- Less than 6 months
- 6-12 months
- 1-2 years
- More than 2 years

. Did your fellowship include training in ESD?

- Yes
- No

. Did you receive any other formal training in ESD through accredited courses or workshops?

- ○ Yes
- ○ No

. How many formal ESD courses or workshops have you attended?

- ○ 1
- ○ 2-3
- ○ 4-5
- ○ >5

. During your ESD training, did you receive mentorship from a senior or experienced ESD endoscopist?

- ○ Yes
- ○ No

. What was the nature of this mentorship? (Select all that apply)

- ☐ Direct hands-on supervision
- ☐ Case discussions and feedback

- ☐ Observation of expert procedures
- ☐ Remote/virtual mentorship
- ☐ Structured proctorship (e.g., formal programme)
- ☐ [ ] Other (please specify)

. During your ESD training, how many procedures did you complete in the following categories?

	0	1-5	6-10	11-20	21-30	>30
Ex vivo ESD procedures	○	○	○	○	○	○
Live pig ESD procedures	○	○	○	○	○	○
Human ESD procedures (assistant-supervised)	○	○	○	○	○	○
Human ESD procedures (primary operator-supervised)	○	○	○	○	○	○

Block 3

. C: Current ESD Practice

. What is the number of ESD practitioners at your centre?

. Approximately how many ESD procedures are performed at your centre per year?

Please choose NA if you don't know or not applicable

	<10	10-20	20-50	50-100	>100	NA
Upper GI ESD	○	○	○	○	○	○
Lower GI ESD	○	○	○	○	○	○

. Approximately how many ESD procedures do you personally perform per year?

Please choose NA if you don't perform ESD in any of the following anatomical regions

	<10	10-20	20-50	50-100	>100	NA
Oesophagus	○	○	○	○	○	○
Stomach	○	○	○	○	○	○
Duodenum	○	○	○	○	○	○
Colon	○	○	○	○	○	○

Please choose NA if you don't perform ESD in any of the following anatomical regions

	<10	10-20	20-50	50-100	>100	NA
Rectum	○	○	○	○	○	○

. How many years have you been performing ESD independently (without direct supervision)?

. Where do you usually perform ESD procedures?

	Operating Room	Endoscopy Unit	Both	I don't perform this type of ESD
Upper GI ESD	○	○	○	○
Lower GI ESD	○	○	○	○

. What type of sedation do you most commonly use for ESD procedures?

	General Anaesthesia	Monitored Anaesthesia Care (MAC) with Propofol	Conscious sedation with Opioids/Benzodiazepines	I don't perform this type of ESD
Upper GI ESD	○	○	○	○
Lower GI ESD	○	○	○	○

Block 4

. D: ESD Techniques and Methods

. Which method do you most frequently use for lesion delineation before ESD? (Select all that apply)

- ☐ White Light Endoscopy (WLE)
- ☐ Narrow Bank Imaging (NBI)
- ☐ Linked Color Imaging (LCI)
- ☐ Blue Light Imaging (BLI)
- ☐ Chromoendoscopy with Indigo Carmine
- ☐ Chromoendoscopy with Acetic Acid
- ☐ Chromoendoscopy with Lugol’s Iodine
- ☐ Confocal Laser Endomicroscopy (CLE)
- ☐ Endoscopic Ultrasound (EUS)
- ☐ Other (please specify): ________

. Which solution do you most frequently use for submucosal injection during ESD? (Select all that apply)

- [ ] Normal Saline
- [ ] Colloids (hydroxyethyl starch and succinylated gelatine)
- [ ] Dextrose water
- [ ] Glycerol
- [ ] Hyaluronic acid (HA)
- [ ] Hydroxypropyl methylcellulose
- [ ] Fibrinogen solutions
- [ ] Eleview and ORISE gel
- [ ] Other (please specify): ________

. Do you add Adrenaline to the injection solution?

- ○ Yes
- ○ No

. Do you add blue dye (such as Indigo Carmine or Methylene Blue) to the injection solution?

- ○ Yes
- ○ No

. Which ESD knives do you most frequently use? (Select all that apply)

- ☐ HookKnife/J (Olympus)
- ☐ Triangle TipKnife/J (Olympus)
- ☐ DualKnife/J (Olympus)
- ☐ FlexKnife (Olympus)
- ☐ HybridKnife I-type (Erbe)
- ☐ HybridKnife T-type (Erbe)
- ☐ FlushKnife BTs (Ball-type) (Fujifilm)
- ☐ FlushKnife NS (Fujifilm)
- ☐ ITknife (Olympus)
- ☐ ITKnife2 (Olympus)
- ☐ ITKnife nano (Olympus)
- ☐ Hybrid-Knife O-type (Erbe)
- ☐ ClutchCutter (Fujifilm)
- ☐ SB Knight (Olympus)
- ☐ Other (please specify): ____

. Which ESD techniques do you most frequently use? (Select all that apply)

- ☐ Conventional (C-ESD)
- ☐ Tunnelling (T-ESD)

- ☐ Pocket-creation method (PCM) including SITE and Double-pocket-butterfly
- ☐ Traction-assisted (T-ESD)
- ☐ Underwater ESD (Saline or Water)
- ☐ Endoscopic intermuscularis dissection (EID)
- ☐ Hybrid ESD
- ☐ [ ] Other (please specify):

. Do you use distal attachment caps?

- ○ Yes
- ○ No

. Do you use traction devices or techniques to assist ESD?

- ○ Yes
- ○ No

. Which traction device or technique do you most frequently use for ESD? (Select all that apply)

- ○ Clip-and-line or Clip-and-thread
- ○ Clip-and-snare
- ○ EndoTrac
- ○ External forceps

- ○ Double endoscope
- ○ Double-channel endoscope
- ○ Additional external devices (AWC, Tracmotion and Endolifter)
- ○ Double-clip traction (Spring or S-O Clip method, rubber band method, multipolar or spider traction method)
- ○ Internal traction wire such as the ProdiGI Traction wire
- ○ Magnetic-assisted traction (magnetic anchor and magnetic bead-assisted)
- ○ Adaptive traction with adjustable force (A-TRACT device)
- ○ [ ] Other (please specify):

. Are you familiar with any experimental robotic-assisted ESD devices?

- ○ Yes
- ○ No

. Which robotic device or system are you familiar with?

[ ]

. Do you think there is a role for robotics in ESD?

- ○ Yes

- No

. Would you be interested in using a robotic-assisted device to perform ESD?

- Yes
- No

. Please rank the following characteristics of a robotic ESD device in terms of usefulness. Consider their impact on improving procedural efficiency, safety, and technical feasibility.

Adjustable/dynamic traction of soft tissue

Independent motion of scope and traction device

Interchangeable instruments

Stabilisation of site

Compatible with current endoscopes

Low footprint

Easy to insert

Works in both Upper and Lower

Block 5

. E: Barriers and Challenges

. How easy is it to secure endoscopy time for ESD at your centre?

- Very easy
- Easy
- Neither easy nor difficult
- Difficult
- Very difficult

. How easy is it to secure the necessary equipment for ESD at your centre?

- Very easy
- Easy
- Neither easy nor difficult
- Difficult
- Very difficult

. How easy is it to secure anaesthesia support for ESD at your centre?

- Very easy
- Easy
- Neither easy nor difficult
- Difficult
- Very difficult

. What is the current waiting time for ESD procedures at your centre?

- <2 weeks
- 2-4 weeks
- 1-3 months
- 3-6 months
- 7-12 months
- >12 months

. Does your centre currently maintain a prospective database of ESD cases and outcomes?

- Yes
- No

. Does your centre offer mentorship or training programmes for consultants with no prior ESD experience?

- Yes, for internal consultants only
- Yes, for external consultants only
- Yes, for both internal and external consultants
- No, but we are considering it
- No, we do not offer ESD mentorship programmes

. Does your centre offer a formal advanced endoscopy fellowship programme, that includes training in ESD, for senior or post-CCT fellows?

- Yes, we offer a structured fellowship programme (nationally/internationally recognised)
- Yes, but it is an informal or locally arranged fellowship
- No, but we are considering starting one
- No, we do not offer advanced endoscopy fellowships

. Do you provide any hands-on training for in-house gastroenterology or surgical trainees?

- Yes
- No

. What are the greatest challenges to the provision of training?

- Heavy workload
- Low ESD caseload
- Lack of suitable cases
- Lack of institutional support
- Lack of formally structured ESD training programme

Powered by Qualtrics
